# Altered Modulation of Silent Period in Tongue Motor Cortex of Persistent Developmental Stuttering in Relation to Stuttering Severity

**DOI:** 10.1371/journal.pone.0163959

**Published:** 2016-10-06

**Authors:** Pierpaolo Busan, Giovanni Del Ben, Simona Bernardini, Giulia Natarelli, Marco Bencich, Fabrizio Monti, Paolo Manganotti, Piero Paolo Battaglini

**Affiliations:** 1 IRCCS Fondazione Ospedale San Camillo, Venice, Italy; 2 B.R.A.I.N. Center for Neuroscience, Department of Life Sciences, University of Trieste, Trieste, Italy; 3 ABC Balbuzie, Turin, Italy; 4 Department of Developmental and Social Psychology, University of Padua, Padua, Italy; 5 Department of Medical, Surgical and Health Sciences, University of Trieste, Trieste, Italy; University of Bologna, ITALY

## Abstract

Motor balance in developmental stuttering (DS) was investigated with Transcranial Magnetic Stimulation (TMS), with the aim to define novel neural markers of persistent DS in adulthood. Eleven DS adult males were evaluated with TMS on tongue primary motor cortex, compared to 15 matched fluent speakers, in a “*state*” condition (i.e. stutterers *vs*. fluent speakers, no overt stuttering). Motor and silent period thresholds (SPT), recruitment curves, and silent period durations were acquired by recording tongue motor evoked potentials. Tongue silent period duration was increased in DS, especially in the left hemisphere (*P*<0.05; *Hedge’s g or Cohen’s d*_*unbiased*_ = 1.054, i.e. large effect size), suggesting a “*state*” condition of higher intracortical inhibition in left motor cortex networks. Differences in motor thresholds (different excitatory/inhibitory ratios in DS) were evident, as well as significant differences in SPT. In fluent speakers, the left hemisphere may be marginally more excitable than the right one in motor thresholds at lower muscular activation, while active motor thresholds and SPT were higher in the left hemisphere of DS with respect to the right one, resulting also in a positive correlation with stuttering severity. Pre-TMS electromyography data gave overlapping evidence. Findings suggest the existence of a complex intracortical balance in DS tongue primary motor cortex, with a particular interplay between excitatory and inhibitory mechanisms, also in neural substrates related to silent periods. Findings are discussed with respect to functional and structural impairments in stuttering, and are also proposed as novel neural markers of a stuttering “*state*” in persistent DS, helping to define more focused treatments (e.g. neuro-modulation).

## Introduction

Developmental Stuttering (DS) is a disruption in the normal rhythm of speech: persons are unable to utter a fluent speech. It begins during childhood and, in some cases, persists in adulthood. DS symptoms are syllable repetitions and blocks, usually at the beginning of sentences/words and in the majority of cases it affects males. It is accompanied by secondary, associated movements/spasms, mainly of facial muscles [1,2]. The cause of DS is not completely clear. It is considered a complex/multifactorial disorder [3,4,5], arising from genetic factors [6], which alter neurologic function [7]. DS may be related to heritable impairments of late fibers myelination [8,9], which may cause the abnormal connectivity observed in DS [10,11]. DS is characterized by neural markers that represent differences in brain anatomy/functioning in comparison to fluent speakers [5,12,13]. Speech-related structures show greater activity in the right hemisphere with respect to homologue areas of the left hemisphere [12], but also greater neural activation in supplementary motor regions [5]. It is not clear if the majority of DS neural abnormalities are a prerequisite for its appearance, or if they are mainly the result of long-term stuttering (comprising attempts to overcome dysfluencies) [14,15]. Overt dysfluency is not essential to differentiate DS brain from fluent individuals (stuttering “*state*” -i.e. DS *vs*. controls- in comparison to a stuttering “*trait*” -i.e. fluency *vs*. dysfluency-) [5,13]. However, DS remains an incompletely understood neurological problem. Speech is a task that requires rapid motor control and its demands may easily be disrupted [16]: thus, dysfluency may be a symptom of a more complex motor syndrome also involving abilities not directly involved in speech [17,18,19,20]. Importantly, DS is characterized by a dopamine-related abnormal functioning of basal ganglia [4,21,22], and it may share characteristics with other basal ganglia-related motor disturbances, such as Tourette’s Syndrome [1] or Parkinson’s Disease, especially when considering timing-related skills [23]. DS improves after administration of antidopaminergic, serotoninergic or GABAergic drugs [24,25,26,27,28,29,30], i.e. substances acting on the excitatory/inhibitory ratio of motor networks that rely also on basal ganglia. Interestingly, previous work suggests slight neural differences between DS males and females [31,32,33], likely also in relation to hormone variability [34]. Neural characteristics of DS have been extensively studied but the potential of non-invasive brain stimulation, and thus the possibility to directly investigate excitatory/inhibitory ratio of neural networks [5], has been underestimated until now. Transcranial magnetic stimulation (TMS) has been used only recently [25,31,35,36,37,38,39,40,41,42,43], especially when considering speech-related motor effectors [37,39,43]. As a consequence, the present work aims to further investigate abnormalities in the excitatory/inhibitory ratio of the primary motor cortex representation of speech muscles (tongue) in persistent DS by using TMS. Considering that neural abnormalities in DS are also present when stutterers are not realizing a speech-specific motor task [38], and/or during rest [31,35,42], in the present work we aimed to investigate the “*basic*” motor activity of speech muscles, in DS males, by measuring TMS indexes, such as motor thresholds, motor evoked potentials (MEPs), and durations of silent periods, in comparison to fluent speakers (some of which will be investigated for the first time in tongue muscles of DS, such as silent period). Measures were recorded aiming to further characterize the aberrant neural functioning in speech-related motor effectors of persistent DS. More specifically, the main objective of the present work is to individuate novel neural markers of a stuttering “*state*”, to better clarify the existing ratio between excitatory and inhibitory motor circuits in DS. As a consequence, data were also correlated with stuttering severity and behavioral/cognitive indexes of DS.

## Materials and Methods

### Participants

A total of 30 participants were recruited for the study, including 14 DS adult males and 16 matched fluent speakers. All DS participants had been developmental stutterers since childhood. Undetected stuttering (and no previous history of DS) was excluded in fluent speakers. All participants had no history of major neurological and/or psychiatric problems. All participants were right-handed Italian native speakers and none were under treatment with psychotropic drugs, such as antidepressants or antipsychotic drugs. Four participants (three DS and one fluent speaker) dropped out due to difficulties in maintaining electrodes on the tongue, TMS discomfort and/or impossibility to evoke reliable responses [44]. Thus, 11 DS adult males (age 24–47 years) and 15 adult fluent speaker males (age 22–42 years) were evaluated and considered in experimental analyses. Of this sample, one DS participant and one fluent speaker were unable to complete the entire procedure due to peripheral stimulation discomfort. Procedures were approved by the Unique Regional Ethical Committee (CERU) of Friuli-Venezia Giulia (referring to the University Hospital of Trieste), and were in accordance with the Declaration of Helsinki (and with recent TMS guidelines) [45]. A screening for evaluating the possible risks related to TMS delivery was also conducted before the experiments [46]. Participants signed a written informed consent before the experiments. Groups were controlled for variables such as sex, age, education, handedness [47], smoking habits [48], migraine [49], musical expertise [50], depressive symptoms (Beck Depression Inventory-II -BDI-II-) [51], and physical activity habits [52,53].

### TMS settings and experimental design

TMS (Medtronic MagPro R30; eight-shaped coil C-B60; biphasic stimulation; antero-posterior direction of the first phase of the current in the coil) was administered to obtain MEPs from tongue muscles on its primary motor cortex representation, in every hemisphere. Participants sat in a relaxed position and they were asked to keep their eyes open during stimulations. Murdoch et al. [54] suggested that an optimal coil direction should be individuated in every participant when investigating tongue motor cortex, but this may add experimental variability. As a consequence, after verifying the presence of reliable MEPs, TMS coil was always maintained on the head by the experimenter, normally at 45° with respect to the inter-hemispheric fissure (coil handle pointing backward). Muscular activity was recorded by four surface Ag/AgCl electrodes on the tongue dorsal part. They were symmetrically placed: two electrodes were about 0.5 cm from the midline, close to the tongue tip, on its right and left side. Remaining electrodes were placed at a distance of about 2 cm, toward the posterior part of the tongue, on external sides. All electrodes were connected to an amplifier, with ground electrode on the right forearm. Positioning of electrodes was checked visually and by electromyography (EMG), considering that a complete rest of tongue is difficult to obtain. A tissue cap was placed on the participants’ head, to individuate the position of the hot-spot on the scalp: it was normally placed about 2 cm ahead and 4 cm laterally, with respect to hand muscles representation) [44]. Tongue representation in primary motor cortex is able to reach both the left and right side of this muscular district [55], and thus measures obtained from contralateral and ipsilateral sides were considered. TMS was delivered by randomized blocks of stimulation to obtain indexes such as motor threshold (MT; asking to maintain 10–20% of tongue maximal muscular activation, visually verified during stimulations), active motor threshold (AMT) and silent period threshold (SPT), asking to maintain 60–70% of tongue maximal muscular activation (dorsiflexion), visually verified on EMG. MT was considered as the stimulation intensity able to evoke MEPs of at least 50–100 μV in half of stimulations. AMT was defined as the stimulation intensity able to evoke a MEP of at least 200 μV in half of the stimulations. SPT [56,57] was defined as the stimulation intensity that was able to evoke a visible and reliable silent period (measured in tens of ms) in half of the stimulations. Motor thresholds were determined by modifying TMS intensity at 1% steps. Silent period durations and latency, recorded maintaining about 60–70% of tongue maximal muscular activation (dorsiflexion) were also bilaterally measured, stimulating at 130% SPT. Silent period duration was measured from the appearance of the MEP until the reappearance of muscular activity [58]. Recruitment curve was recorded by stimulating at 110%, 125%, and 140% MT, maintaining lower tongue activation (see MT). Tongue MEPs were considered reliable when responses showed latencies comprised between about 6–13 ms [44]. Two adjunctive EMG channels were used to evaluate artifacts: the first channel evaluated peripheral TMS responses, such as jaw displacement due to peripheral nerve stimulation (Ag/AgCl electrodes placed on the jaw), while the second was used to evaluate TMS-evoked eye blinks (Ag/AgCl electrodes placed around eyes). We also registered MEPs from first dorsal interosseous (FDI) muscle in DS, by stimulating the left and the right primary motor cortex and recording contralateral responses using Ag/AgCl electrodes placed in a tendon-belly montage. AMT and SPT were recorded, asking participants to maintain a muscular contraction of about 30–40% of maximal activation, as well as silent period durations (measured as above indicated), stimulating at 150% SPT. This was realized to compare silent period data obtained from hand muscles of persistent DS males [31], and data obtained from tongue, with stuttering severity in the same sample of participants, considering the existence of neurophysiological heterogeneity in DS [14,15]. TMS coil was maintained by the experimenter at 45° with respect to the inter-hemispheric fissure, with the handle pointing backward. EMG data before TMS delivery were also analyzed to verify if the groups were homogeneous when considering spontaneous tongue EMG activity, bilaterally. In this case, 20 ms before TMS delivery were considered in recruitment curve data, and 60 ms in silent period (duration and latency) data. EMG was acquired by using a sampling rate of 8000 Hz, visualized by a digital band-pass filter of 20–2000 Hz. Tongue silent period (duration and latency) was obtained by averaging about six consecutive stimulations, in every hemisphere. The same was done for every stimulation intensity (in every hemisphere) for recruitment curve. When considering FDI silent periods in DS, about six stimulations were averaged, in every hemisphere. Experimental design was implemented to minimize muscular fatiguing; TMS was delivered by applying random interstimulus intervals of about 2–6 seconds.

### Behavioral measures

Severity of stuttering was evaluated in DS by using the Stuttering Severity Instrument-4 (SSI-4) [59]. A speech sample was acquired from DS participants, measuring percentages of stuttered syllables, the severity of physical concomitants, and the duration of the longest blocks. Both groups were administered an Italian version of the BigCAT Questionnaire [60], a self-evaluation of speech-associated attitudes (negative or positive) adapted to DS (35-items). The Italian version of the Cognitive Behavioral Assessment (CBA) 2.0 scale [61] was also administered: it is a battery of self-administered questionnaires useful for investigating personality characteristic, emotional adjustment, and psychological status, evaluating whether groups differ in behavioral/cognitive states/traits. Data were correlated with neurophysiologic indexes.

### Statistical analysis

Motor thresholds were expressed in percentages with respect to maximal TMS output. Recruitment curve data were expressed as peak-to-peak amplitudes (μV), latencies (ms), and areas under the curve (V/sec). Silent period durations and latencies were expressed in ms. EMG data (pre-TMS), were evaluated considering areas under the curve (V/sec). Raw EMG data (obtained from TMS and pre-TMS recordings) were always considered for analyses, i.e. data were not rectified and/or averaged between conditions before calculations, in order to limit data handling. Data from cognitive/behavioral measures (SSI-4, BigCAT, CBA 2.0) were reported as interval measures. Statistical analyses were performed by mixed model analysis [62,63]. When considering TMS, factors were groups (stuttering *vs*. fluent speakers), stimulated hemispheres (left *vs*. right), and, when considering silent period durations, silent period latencies, and recruitment curve, also side of the tongue (left *vs*. right). When considering recruitment curve, also stimulation intensities were considered (110%, 125%, 140% MT). Main effects and interactions among factors were investigated. Suitable degrees of freedom for the mixed model analysis were approximated by considering sample sizes, and subtracting free parameters. Pre-TMS EMG data were analyzed by considering groups, stimulated hemispheres, tongue sides, and stimulation intensities (when appropriate). In post-hoc analyses, cognitive/behavioral variables, and analyses of homogeneity (i.e. age, handedness, education, musical expertise, smoke habits, migraine, and physical activity), data normality was evaluated by the Shapiro-Wilk Test. Homogeneity of variance was also verified in between-groups comparisons. Differences in normally distributed data were assessed by Student’s *t*-test (Welch’s *t*-test in not homogenous data; *t*), while non-parametrical tests were performed in not normally distributed data (Mann-Whitney or Wilcoxon rank sum test; *Z*). Categorical data (physical activity) were evaluated by using a Chi-square test (Yates correction for low frequencies). Neef at al. [37] suggested that neurophysiologic measures may not always be independent with an unknown real degree of correlation, making classical corrections for multiple comparisons unfeasible. Moreover, no formal consensus has been reached on this topic [64,65,66]. As a consequence, we decided to report here raw, uncorrected, values, applying a false discovery rate method on a family-wise basis (i.e. behavioral data, motor thresholds data based on MEPs, recruitment curves, silent period data, pre-TMS EMG data; significant values that did not survive are accompanied by (*)) [67], on pairwise, between/within groups post-hoc comparisons. An estimate of the effect sizes was also performed in significant two-means comparisons, depending on data normality and homogeneity, and between/within-participants comparisons (absolute values; *Hedge’s g or Cohen’s d*_*unbiased*_, *r*, *Glass’s delta*, *Φ*, *d*: 0.2<*d*_*unbiased*_, *delta*, *d*<0.5 small effect size; 0.5<*d*_*unbiased*_, *delta*, *d*<0.8 medium effect size; *d*_*unbiased*_, *delta*, *d*>0.8 large effect size; 0.1<*r*<0.3 small effect size; 0.3<*r*<0.5 medium effect size; *r*>0.5 large effect size) [68,69,70,71,72,73,74]; in non-parametric comparisons both *r* and the parametric counterparts are reported, to allow a more complete evaluation of effects. In this context, “*a priori*” statistical power (about 80%) calculations, mainly based on hypothesized differences between groups in silent periods and recruitment curves, justified sample sizes (considering also possible deviations from normality in data). Finally, a correlation analysis was also performed to evaluate relations between SSI-4, TMS indexes, and behavioral/cognitive data, in DS and fluent speakers, by Pearson’s correlation (*r*; in normally distributed data), and by Spearman’s correlation (in not normally distributed data; in alternative, Gamma statistic was used when more appropriate -i.e. when tied observations were also present; *Γ*). A *P*<0.05 was considered significant (two-tailed assumption; *P*<0.1: trend toward significance).

## Results

### Behavioral measurements

No significant differences were evident between groups when considering measures evaluating group homogeneity in factors such as age, education, handedness, depressive symptoms (BDI-II; this was confirmed also when separately evaluating cognitive/somatic symptoms, statistics not reported), smoking habits, musical expertise, migraine, and physical activity (see Table 1). SSI-4 showed that DS ranged from “*very mild”* to a “*severe”* level. BigCAT showed a higher negative attitude toward speech situations in DS than controls (effect size: *r* = 0.815 -*Hedge’s g or Cohen’s d*_*unbiased*_ = 2.876-; see Table 1 for significance). When considering CBA 2.0, DS group resulted significantly different with respect to fluent speakers in subscales such as the EPQ/R-N scale (*Hedge’s g or Cohen’s d*_*unbiased*_ = 0.878; higher levels of emotional lability in DS), the QPF/R scale (*r* = 0.567 -*Hedge’s g or Cohen’s d*_*unbiased*_ = 1.337-; higher tendency of psychophysiological disturbances in DS), and in the IP/PH scale (*r* = 0.612 -*Hedge’s g or Cohen’s d*_*unbiased*_ = 1.514-; higher levels of phobia in DS). Scores obtained from DS and fluent speakers always resulted under the threshold for psychopathological disturbance (≥95^th^ percentile). On a singular participant level, two fluent speakers had values ≥95^th^ percentile, while six DS had values ≥95^th^ percentile. The main findings are reported in Tables 1 and 2.

**Table 1:** Summary of the characteristics of the DS and fluent speakers groups.

*Group characteristic/Exp*. *Group*	Stuttering	Fluent speakers	Statistics	P
**Age**	32.8 (9.0)	29.5 (5.6)	Z = 0.628	0.55
**Education**	17.5 (3.9)	15.7 (2.3)	Z = 1.079	0.29
**Handedness**	83.2 (12.8)	85.9 (12.3)	*t*_24_ = 0.562	0.58
**Beck Depression Inventory-II**	5.1 (5.0)	3.1 (4.0)	Z = 1.079	0.29
**Smoking**	0.29 (0.5)	0.20 (0.4)	Z = 0.805	0.48
**Musical Expertise**	0.31 (0.5)	0.18 (0.4)	Z = 1.244	0.24
**Migraine**	0.02 (0.1)	0 (0)	Z = 1.677	0.42
**Physical Activity**	5/6	12/3	χ^2^ (Yates correction)	0.16
**BigCAT**	**24.9 (10.3)**	**3.7 (3.4)**	**Z = 4.154**	**<0.001**
**EPQ/R-N scale (CBA 2.0)**	**60.4 (24.4)**	**36.3 (28.0)**	***t*_24_ = 2.277**	**0.031**
**QPF/R scale (CBA 2.0)**	**63.4 (26.5)**	**26.4 (27.0)**	**Z = 2.889**	**0.003**
**IP/PH scale (CBA 2.0)**	**51.8 (20.2)**	**25.3 (14.2)**	**Z = 3.120**	**0.001**

Data obtained from the main characteristics evaluated to match experimental groups. Data regarding smoke habits, musical expertise, migraine have been evaluated on a scale basis, while physical activity has been evaluated on a categorical basis. Data obtained from speech attitudes evaluation (BigCAT) and data resulted significantly different in cognitive/behavioral evaluation (CBA 2.0) are also reported. Data are reported indicating mean and standard deviation in brackets. Significant differences between groups are marked in bold.

**Table 2:** Stuttering Severity Instrument-4 evaluation of DS participants.

*Stuttering participant/SSI-4 indexes*	SSI-4 value	SSI-4 percentile	SSI-4 category
a	18	12–23	*Mild*
b	31	61–77	*Moderate*
c	25	41–60	*Moderate*
d	30	61–77	*Moderate*
e	32	78–88	*Severe*
f	23	24–40	*Mild*
g	12	1–4	*Very mild*
h	32	78–88	*Severe*
i	31	61–77	*Moderate*
j	36	89–95	*Severe*
k	13	5–11	*Very mild*

Stuttering Severity Instrument-4 scores, in every DS participant, are reported.

### Motor thresholds

**Fig 1:**
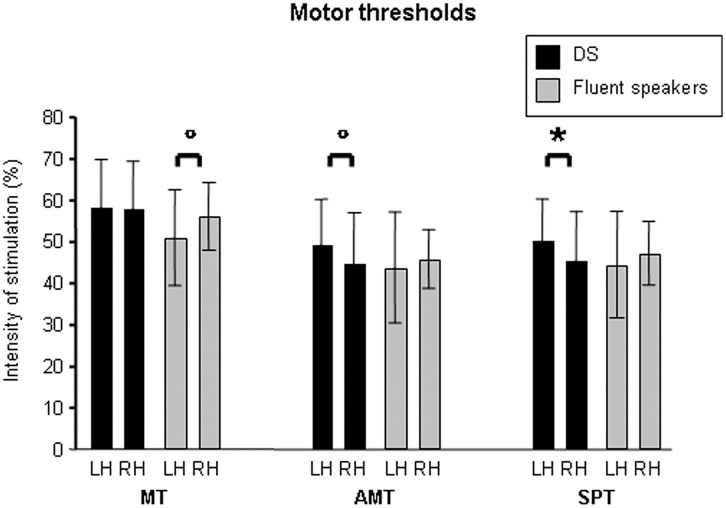
Motor thresholds in the stuttering and fluent speakers groups. Data obtained for MT, AMT, and SPT are reported for DS and fluent speakers. Statistically significant comparisons are indicated with an asterisk, while marginally significant comparisons are indicated with a circle.

When considering TMS data and, more specifically, motor thresholds comparisons, statistics showed a marginal difference between groups and stimulated hemispheres in MT (overall significance of the model, *P* = 0.063; groups *x* stimulated hemispheres: *t*_22_ = -1.845, *P* = 0.079). This was supported by the fact that the fluent speakers’ left hemispheres had lower MT with respect to their right one (*t*_14_ = 2.414, *P* = 0.030(*); *d* = 0.475), while this difference was not highlighted in DS. When considering AMT, there was a marginal difference between groups and stimulated hemispheres (overall significance of the model, *P* = 0.065; groups *x* stimulated hemispheres *t*_22_ = -1.836, *P* = 0.081). More specifically, left hemisphere AMT resulted higher with respect to the right hemisphere AMT in DS (*t*_9_ = 1.958, *P* = 0.082(*); *d* = 0.411). Finally, when considering SPT, there was a significant difference between DS and fluent speakers when considering the interaction between groups and stimulated hemispheres (overall significance of the model, *P* = 0.020; groups *x* stimulated hemispheres: *t*_22_ = -2.374, *P* = 0.027). Findings suggest higher SPT in the left hemisphere of DS participants with respect to their right one (*t*_9_ = 2.388, *P* = 0.041; *d* = 0.466). The main findings are summarized in Table 3 and Fig 1.

**Table 3:** Summary of the main findings obtained by TMS.

*Neurophysiologic index/Exp*. *Group*	Stuttering LH	Stuttering RH	Fluent speakers LH	Fluent speakers RH
**MT (%)**	58.3 (11.3)	57.8 (11.4)	*50*.*8 (11*.*5)*	*55*.*9 (8*.*1)*
**AMT (%)**	*49*.*1 (10*.*8)*	*44*.*7 (11*.*9)*	43.6 (13.4)	45.6 (7.1)
**SPT (%)**	**50.2 (9.9)**	**45.4 (11.7)**	44.1 (12.9)	47 (7.7)
**110% MT amplitude (μV)**	*184*.*5 (140*.*6)*	*103*.*5 (59*.*7)*	148.2 (109.3)	152.0 (171.4)
	*153*.*3 (127*.*5)*	*110*.*8 (62*.*9)*	138.9 (122.4)	147.4 (129.5)
**125% MT amplitude (μV)**	336.0 (248.0)	315.7 (230.0)	298.3 (249.0)	205.8 (144.7)
	340.1 (205.4)	350.2 (255.5)	262.4 (250.9)	248.7 (184.5)
**140% MT amplitude (μV)**	469.7 (248.9)	465.1 (371.4)	559.6 (403.9)	362.8 (175.1)
	426.9 (188.5)	427.1 (332.4)	524.6 (422.0)	436.2 (264.6)
**110% MT area (V/sec)**	772.7 (472.2)	563.7 (569.4)	672.2 (551.6)	723.0 (832.5)
	662.6 (517.0)	621.9 (679.2)	611.3 (508.0)	605.8 (668.6)
**125% MT area (V/sec)**	*1495*.*8 (910*.*7)*	*1555*.*1 (996*.*7)*	*1472*.*1 (1609*.*9)*	*1054*.*9 (1004*.*1)*
	*1724*.*1 (977*.*8)*	*1735*.*1 (1036*.*3)*	*1319*.*0 (1551*.*1)*	*1092*.*7 (985*.*4)*
**140% MT area (V/sec)**	2388.5 (1356.8)	2392.9 (1919.1)	2794.4 (2328.3)	1940.7 (1331.1)
	2382.9 (1306.1)	2173.8 (1516.4)	2646.5 (2376.4)	2264.1 (1539.3)
**110% MT latency (ms)**	10.0 (1.7)	10.2 (0.9)	9.5 (1.1)	9.9 (1.1)
	10.1 (1.6)	10.1 (1.8)	10.1 (1.4)	10.3 (1.1)
**125% MT latency (ms)**	10.1 (1.9)	10.1 (1.0)	9.7 (1.0)	9.9 (1.3)
	10.1 (1.5)	9.8 (1.5)	9.8 (1.5)	10.0 (1.5)
**140% MT latency (ms)**	9.7 (1.5)	10.5 (1.0)	9.3 (1.3)	10.0 (1.2)
	9.5 (1.1)	10.0 (1.6)	9.4 (1.6)	9.7 (1.2)
**Silent period duration (ms)**	**51.6 (7.6)**	50.2 (9.1)	**44.1 (8.5)**	46.7 (9.6)
	**51.1(4.9)**	46.8 (11.3)	**42.8 (7.6)**	47.3 (13.7)
**Silent period latency (ms)**	9.7 (1.3)	9.9 (1.2)	9.4 (1.7)	9.5 (1.1)
	10.1 (1.6)	9.9 (1.9)	10.1 (1.7)	9.6 (1.1)

Mean values are accompanied by standard deviations in brackets. Data are reported for right/left side of the tongue when considering amplitudes, areas and latencies of recruitment curve data, silent period durations and latencies. Significant comparisons are reported in bold, marginal differences in *italic*; LH = left hemisphere, RH = right hemisphere.

### Recruitment curves

When considering recruitment curves, MEPs amplitudes (*P*<0.001) show a general effect related to intensity of stimulation (*t*_23_ = 6.395, *P*<0.001; *t*_23_ = 4.260, *P*<0.001). An effect was evident in the interaction between groups, stimulated hemispheres, and intensities of stimulation (*t*_19_ = 2.111, *P* = 0.048), showing marginal difference in DS, when comparing MEPs obtained in the two hemispheres and stimulating at 110% MT (left hemisphere MEPs higher of right hemisphere MEPs; *Z* = 1.956, *P* = 0.05(*); *r* = 0.590 -*d* = 0.588-). When considering MEPs areas (significance of the overall model: *P*<0.001), an effect related to intensity of the stimulation was evident (*t*_23_ = 6.040, *P*<0.001; *t*_23_ = 3.760, *P* = 0.001). A marginal effect related to the interaction between groups and intensity of stimulation was also evident (*t*_21_ = -1.745, *P* = 0.096), suggesting a possible difference between DS and fluent speakers when stimulating at 125% MT (*Z* = 1.946, *P* = 0.054(*); *r* = 0.382 -*Hedge’s g or Cohen’s d*_*unbiased*_ = 0.389-; higher MEPs areas in DS). Fluent speakers showed a significant negative correlation of MEPs areas (obtained when stimulating the right hemisphere at 125% MT) with physical activity (*Γ* = -0.78). No differences were evident in MEPs latencies. Pre-TMS EMG analyses related to recruitment curve are reported in the Supporting Information (S1 File). The main findings are summarized in Table 3, Table A and Fig A in S1 File.

### Silent period durations

**Fig 2:**
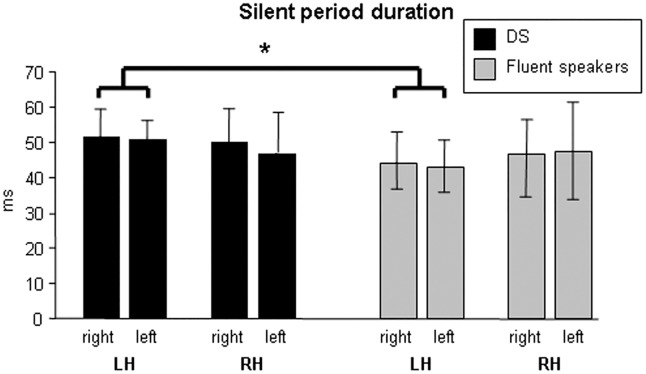
Silent period durations obtained in the stuttering and fluent speakers groups. Significant differences are indicated with an asterisk. Data are reported by considering also the right/left side of the tongue. LH = TMS administered on the left hemisphere; RH = TMS administered on the right hemisphere.

**Fig 3:**
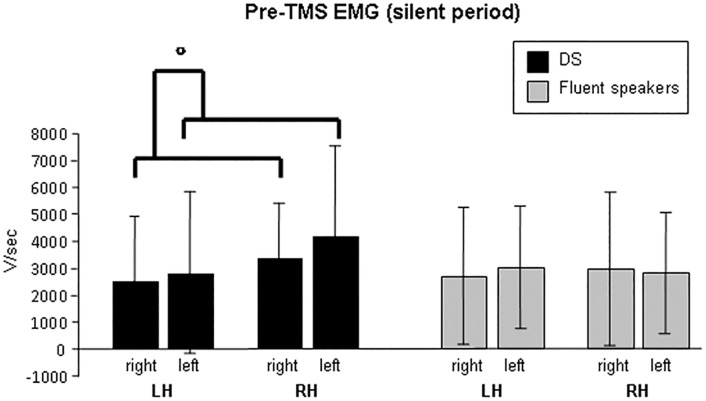
Pre-TMS EMG data obtained in the two groups during sustained tongue contractions requested for the silent period TMS protocol. About 60–70% of maximal muscular activation was requested. Marginally significant comparisons are indicated with a circle. Data are reported by considering also the right/left side of the tongue. LH = TMS administered on the left hemisphere; RH = TMS administered on the right hemisphere.

When considering silent period durations, statistics resulted significant (overall significance of the statistical model, *P* = 0.044), suggesting differences in the interaction between groups and stimulated hemispheres (*t*_22_ = -2.257, *P* = 0.034). More specifically, silent period durations were longer when stimulating tongue motor cortex of the left hemisphere in DS, with respect to fluent speakers (*t*_23_ = 2.651, *P* = 0.014; *Hedge’s g or Cohen’s d*_*unbiased*_ = 1.054). Analysis of silent period latencies did not revealed significant differences. When considering pre-TMS EMG data related to silent period, recorded side of the tongue resulted significantly different (*t*_24_ = -2.403, *P* = 0.025), suggesting that the right side is generally more activated, with respect to the left side, during spontaneous and sustained contractions before TMS delivery, in both groups (*Z* = 1.841, *P* = 0.066(*);*r* = 0.361 -*d* = 0.211-). The interaction between groups and recorded side of the tongue resulted marginally significant (*t*_22_ = 2.023, *P* = 0.055), suggesting a greater difference in DS (with respect to fluent speakers) in the spontaneous and sustained pre-TMS EMG activity, when comparing the tongue right side versus the left one (*Z* = 2.845, *P* = 0.004; *r* = 0.858 -*d* = 0.429-). The main findings are summarized in Table 3, Table A in S1 File, Figs 2 and 3, and Fig B in S1 File.

### Correlation analysis

**Fig 4:**
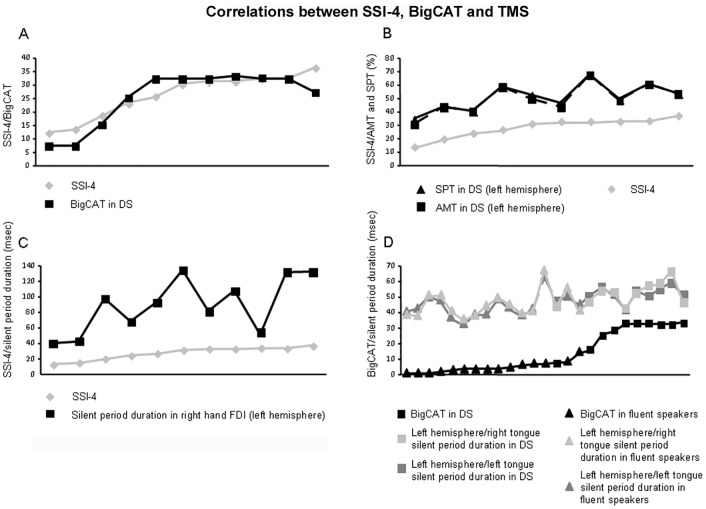
Significant correlations between TMS data and stuttering severity. Main significant findings obtained from correlation analyses between SSI-4, BigCAT, and TMS are reported. Participants are reported on the *x*-axis. Positive correlation between SSI-4 and BigCAT in DS are reported in (A); positive correlation between SSI-4, tongue AMT and SPT (obtained when stimulating the left hemisphere) in stuttering are reported in (B); positive correlation between SSI-4 and silent period durations, obtained from the right FDI muscle when stimulating the left hemisphere motor cortex in DS are reported in (C); positive relation between BigCAT and silent period durations recorded from tongue muscles (left/right side) when stimulating the left hemisphere motor cortex in all participants, is reported in (D).

Here only significant results (*P*<0.05) related with the principal aim of the manuscript will be reported (main relations between TMS, SSI-4 and BigCAT are here reported; remaining correlations are reported in S1 File), without reporting irrelevant correlations (e.g. correlations between motor thresholds and TMS data) or marginal correlations (*P*<0.1). When considering SSI-4, a positive relation with BigCAT is evident (*Γ* = 0.63; participants with higher DS severity perceive more negative attitudes toward speech situations). When considering TMS data, SSI-4 showed a positive relation with tongue AMT and SPT of the left hemisphere (AMT: *r* = 0.68; SPT: *r* = 0.69). Similarly, silent period durations of the right FDI muscle (i.e. when stimulating the left hemisphere) were positively related with SSI-4 (*r* = 0.68), and a positive relation was highlighted between SSI-4, bilateral AMT and SPT of FDI muscles (left hemisphere AMT: *r* = 0.62; right hemisphere AMT: *r* = 0.75; left hemisphere SPT: *r* = 0.62; right hemisphere SPT: *r* = 0.75). A positive relation was evident in all participants (DS and fluent speakers), between bilateral tongue SPd, obtained stimulating the left hemisphere, and the BigCAT (tongue right side: *Γ* = 0.41; tongue left side, *Γ* = 0.43). The main findings are summarized in Fig 4.

## Discussion

### Summary of findings

In the present work, the main findings suggest that longer silent period durations may be evident in DS, when recording from tongue muscles, even when no overt dysfluency is present and during no speech tasks, especially when stimulating the left hemisphere motor cortex. Moreover, DS had higher SPT in the left hemisphere with respect to the right one. A similar but less defined pattern is evident when considering AMT (see also preliminary cases report of Barwood et al. [36]) while MT (obtained during lower muscular activation) had a higher asymmetry in fluent speakers (left hemisphere more excitable than the right one),which was not evident in DS (compare with [39]). TMS data (tongue and hand muscles), such as motor thresholds and SPd, showed positive correlations with SSI-4, as well as with DS behavioral/cognitive indexes (some of which resulted more elevated in DS). A positive relation was evident in DS between left hemisphere AMT and SPT, recorded from the tongue, and SSI-4, as well as between left hemisphere silent period durations of the tongue and BigCAT (in all participants; BigCAT was positively related to SSI-4). Silent period durations of the right FDI (i.e. stimulating the left hemisphere) were positively related with SSI-4, and a positive relation was evident between SSI-4 and bilateral AMT and SPT of FDI. Pre-TMS EMG measures mainly showed that DS could be related to an imbalance in realizing sustained and volitional muscular contractions through the projections toward the tongue right side (lower activity) with respect to those directed to the left side (higher activity), and to a higher variability (see also S1 File). DS showed significant negative attitudes toward speech situations and higher levels of emotional lability, phobia, and psychophysiological disturbances. In the following, data will be discussed with respect to available evidence in literature, trying to further clarify the functioning of neural motor patterns in persistent DS.

### The delicate (dis)equilibrium between neural networks in DS

The present findings suggest that persistent DS is related to a delicate interplay between different cortical/intracortical mechanisms involved in the functioning of tongue motor cortex, thus influencing cortico-bulbar responses, even when no overt stuttering is evident. Motor thresholds and recruitment curve data point toward the confirmation of the presence of lower left hemisphere cortico-bulbar excitability in DS, in contrast to higher activity in homologue structures of the right one [12,13], with respect to fluent speakers. On the other hand, lower inhibitory responses in the left hemisphere of DS, when considering SPT, was also evident. This index represents the functionality of intracortical networks (see below, silent period discussion section), and suggests the presence of a continuous interplay between excitatory and inhibitory motor mechanisms in DS, influencing the final speech and not strictly speech related motor output, likely looking for homeostasis (in this case, trying to avoid speech dysfluencies). Pre-TMS EMG differences (see also S1 File) may also be interpreted as being due to the presence of a lower (and more variable) activity in tongue motor regions of the left hemisphere with respect to the right hemisphere, in stuttering. Previous evidence on this is conflicting, possibly also showing no differences in EMG activity between DS and fluent speakers [37,39]. DS is related to an aberrant language brain lateralization, especially of motor outputs needed for correct speech execution [32,75,76,77]. These neural patterns may be related to mechanisms that help compensate for an aberrant transmission among inferior frontal regions and speech sensorimotor cortices [10,11]: this is supported by evidence showing an inverse correlation between right hemisphere neural activity and indexes of stuttering severity [78,79,80], as well as no evidence of this augmented activity in DS children [81]. In the end, this may be related to imbalanced wiring in DS [5]. As a consequence, motor planning/execution of motor tasks in speech muscular areas need the punctual integration of excitatory and inhibitory neural signals. Indeed, DS shows less and/or different neural changes during motor planning in both speech- and non-speech-related tasks in frontal, temporal, and sub-cortical regions [82,83]. The correct functioning of this system may be related to signaling and connections from left frontal regions [10,11,84], and also from the sub-cortical basal ganglia system [3,85].

### DS and impairments in white matter and in the basal ganglia system: relations with present findings and insights from previous TMS studies

White matter abnormalities are indicated as one of the fundamental neural markers of DS [5,8,10,11,13]: they are widespread and evident in cortical and sub-cortical networks, comprising structures such as corpus callosum, superior longitudinal fasciculus, fibers of chorona radiata, cortico-spinal and cortico-bulbar tracts, frontal regions (e.g. premotor and sensorimotor regions), left angular gyrus, arcuate fasciculus, and thalamo-cortical circuits [5,8,81,85,86,87,88,89,90,91]. They are preferably evident in the left hemisphere [8,10,11,80,84,86,92]. With respect to the present findings, Sommer et al. [10] reported less white matter in the left operculum of DS in correspondence of the tongue and larynx sensorimotor representation (see also [81]). Similarly, Connally et al. [89] reported weaker connectivity in left cortico-bulbar and cortico-spinal tracts in DS (versus augmented connections in the right hemisphere), and reduced white matter in the bilateral arcuate fasciculus; higher stuttering severity was related with higher connectivity in the left cortico-bulbar fibers. Jäncke et al. [93] showed increases in white matter in right hemisphere networks in DS adults, comprising the inferior frontal gyrus and face/mouth motor representations. Watkins et al. [11] showed less white matter in bilateral premotor/motor regions in DS, as well as in fibers of inferior frontal regions that are involved in speech motor aspects (see also [80]); lower neural activity in sensorimotor and inferior frontal regions in DS was also reported. Chang et al. [80,81] showed that DS adults and children have lower white matter levels in the left superior longitudinal fasciculus, which transmits information to motor structures and left inferior frontal regions. Interestingly, Kemerdere et al. [94] suggest that the stimulation of the left frontal aslant tract induce stuttering in fluent speakers: the bilateral frontal aslant tract has an increased mean diffusivity in persistent DS, and a negative correlation between fluency and mean diffusivity in the left tract was evident. The frontal aslant tract connects inferior frontal regions with the supplementary motor complex, playing a role in speech motor organization [90,95]. DS related neural fiber impairments are also in direct relationship with sub-cortical neural targets, such as basal ganglia. In fact, DS could be also viewed as a movement disorder related to basal ganglia dysfunction [3,4,23], and to dopaminergic hyperactivity in these regions [21,22]. Intracortical neural circuits, and consequently cortico-spinal/cortico-bulbar activity, may be influenced by dopamine modulation [96,97,98]. In fact, basal ganglia dysfunction in DS may influence cortical functioning by means of cortico-striatal-thalamo-cortical loops [19]. Stuttering severity and dysfluencies have been showed to be related with basal ganglia activity [5,11,99,100,101], before but not after fluency-inducing treatments [99]. Supplementary motor area (part of the cortico-striato-thalamo-cortical loop) [19] hyperactivity is consistently found in DS [5,13], which likely influenced motor cortex activity [102]. Considering its relation with basal ganglia dysfunction, stuttering may be considered as a timing impairment in neural networks that support volitional, self-paced (preferably speech-related) motor acts, relying on dysfunction of left hemisphere motor structures, with a sub-optimal connectivity of basal ganglia-thalamo-cortical and motor neural networks [83,92], also indirectly involving speech-related muscular districts. Interestingly, present and previous TMS findings in DS are consistent with the above reported white matter abnormalities. Only few studies investigated DS by TMS. Moreover, previous work has often concentrated on non-speech-related aspects of DS motor system, because of the challenging methods needed to record TMS-evoked potentials from speech muscles. This led to the definition of DS as a more general motor impairment [25,31,35,38,42]. DS abnormal motor functioning is also evident when no overt stuttering is present, helping to disentangle “*basic*” neural excitability in DS, and its modulation during speech- or non-speech-related tasks [25,31,35,37,38,39,42]. Abnormal ratios of neural activity have been shown between left and right hand motor cortex [35] and with respect to fluent speakers, both during rest [31,42] or rhythm-related motor tasks [38]. Intracortical excitability seems to be normal in DS when considering hand motor representations [41,42], even if it responds to selective serotonin re-uptake inhibitors aimed at reducing stuttering [25]. On the other hand, abnormal patterns of excitability affect intracortical (and cortico-bulbar) networks of speech muscles in DS [36,37,39,40]: a weaker inhibition in the right hemisphere (associated with delayed inhibition of intracortical circuits in both hemispheres), and a bilaterally reduced facilitation was evident in DS, accompanied by steeper stimulus-response curves (preferably during muscular activation of the same districts), especially in the right hemisphere [36,39,40]. Moreover, the lack of left hemisphere facilitation of tongue MEPs during speech was evident, suggesting an asymmetry controlling speech motor planning in motor cortex [37]. Parts of these findings correlated with stuttering severity. The lack of a left hemisphere facilitation of MEPs during speech [37], as well as the evidence of lower neural activity in regions such as larynx motor cortex [5], may be related with pre-existent abnormal excitatory/inhibitory ratios in intracortical circuits modulating DS motor outputs, demonstrated here by findings related to silent period durations and SPT. Indeed, the present findings may be also viewed as the missing link of a more complex picture in which intracortical motor circuits play a central role, even in a more general stuttering “*state*” condition. Present correlations between stuttering indexes, AMT, SPT, and silent period durations of both hemispheres in speech- and not strictly speech-related muscular districts (see also [31]) support these suggestions. These observations may help to understand how different neural substrates may be mutually related to maintain the disorder, also in adulthood. One of the possible mechanisms of this relationship is highlighted in the next section.

### DS and cortical silent period: possible relations with basal ganglia and white matter abnormalities

DS motor thresholds and recruitment curves have also been investigated in previous TMS studies in both speech (and not strictly speech-related) muscles with a certain level of agreement [25,31,35,36,37,38,39,40,41,42,43]. Here, we extend those observations, considering that silent periods have been not extensively studied in DS, especially in DS speech muscles, such as the tongue. Silent period is considered a temporary suppression of voluntary muscle activity, after depolarization of motor neuronal cells by TMS. Mechanisms underlying silent period are still not completely understood (see [57]) and, thus, interpretation of results may be not simple. Silent period depends on both spinal and cortical mechanisms (likely interruptions of the cortical drive), resulting from the influence of intracortical inhibitory cells on the motor cortex [103,104]: it is an index of intracortical inhibition modulated by GABAergic activity of interneurons that synapse with neurons of pyramidal tracts [105]. In DS, it has been reported that not directly speech-related silent period may have a role in the effects of drugs that help to manage stuttering [25]. Rogić Vidaković et al. [40] showed differences in the silent period of DS hand muscles in both hemispheres. The present, and previous, findings [31] suggest that silent period may be differently related with indexes of stuttering severity in both speech- and not strictly speech-related muscles. As a consequence, this TMS index may have an important role in DS neurophysiology, likely representing a neural marker of DS intracortical inhibition and functioning, especially in the motor structures of left hemisphere (please note, that the different intracortical TMS indexes until now reported in DS have mainly been obtained from paired pulse protocols, with different evidence, such as for example the lacking of a clear correlation with stuttering severity; see [39,41,42]). Speculatively, the here reported lengthened silent period can be related, for example, to the consequences of a decrease in tonic excitation modulated by afferent pathways to motor cortex, as a result of the widespread white matter impairments evident in DS, favoring a prolonged GABA-mediated inhibition on pyramidal cells. It might be related to a general lack of inhibition of neurons in motor cortex, again resulting in an over-activation of GABAergic interneurons (also as a consequence of the DS abnormalities in basal ganglia-thalamo-cortical circuits), favoring plastic neural mechanisms looking for a sustainable equilibrium between excitatory/inhibitory signals in the brain. Interestingly, a lengthened SP in the affected hemisphere of stroke patients was associated with motor deficits such as movement initiation and inability to maintain constant force levels, fitting with present evidence, while clinical improvements were related to shortened silent periods [106], also in DS [25]. Classen et al. [106] suggested that motor dysfunctions may be related to the hyperactivity of inhibitory mechanisms in the cortex, and an increased silent period may result from damage to different input pathways of the motor cortex, as it seems to be the case in DS. A diminished and/or augmented activity of inhibitory interneuronal systems may result in abnormalities of the (motor) neuronal networks, considering that they should target and modulate (motor) neural activity (by means of lateral inhibition), as seems to be the case in a series of other basal ganglia related motor disturbances such as Parkinson’s Disease [96], Tourette’s Syndrome [107], and dystonia [108].

### DS sub-groups from a neurophysiologic point of view?

Present correlation analyses showed that pre-TMS EMG data (see S1 File) were poorly correlated in DS with respect to fluent speakers, confirming higher variability in DS when a motor task is required [109,110,111]. In general, differences with previous reports may be due to methodological issues or related to the presence of different neurophysiological profiles in persistent DS, i.e. in adults that modeled their brain patterns during attempts to manage dysfluencies [14,15]. Indeed, when recording from FDI muscles, a positive relation is evident between stuttering severity, left hemisphere silent period duration, and bilateral AMT/SPT: higher stuttering degree was associated with higher activity in intracortical inhibition networks of left hemisphere and with particular excitatory/inhibitory ratio of neural activity also in motor regions not directly involved in speech control. This supports Busan et al.’s [31] observations that DS males showed a negative correlation between silent period durations obtained when stimulating right hemisphere FDI representation, and stuttering severity. This supports the idea that different DS groups may show slight differences in neurophysiologic profiles, pointing toward a generalized left hemisphere motor inhibition, counter-parted by higher right hemisphere activity in homologue brain regions. Moreover, it may sustain the idea that DS may be the only overt symptom of a more general motor disorder (see [31]). In general, different possible DS sub-divisions have been proposed, on the basis of neural and genetic factors [112], effect of pharmacological agents (see [3]), neural and genetic differences between DS males and females [31,32,33,113], anxiety levels, and co-morbidities [114]. Indeed, the present findings sustain the idea that DS may be related to higher levels of emotional lability [115,116], higher tendencies toward psychophysiological disturbances, and higher levels of phobia, likely related to DS social implications (see [117,118]; see also further additional correlations reported in S1 File). In fact, here we report that a negative attitude toward speech situations is evident in DS (please note also the positive correlation between SSI-4 and BigCAT scores; see [119]); BigCAT (negative speech attitudes in DS) also positively correlated with bilateral AMT and SPT obtained from hand muscles (see S1 File).

### Methodological issues and limitations of the study

The present work has some limitations. For example, recordings from the tongue are methodologically challenging and uncomfortable, and as a consequence a limited amount of data can be obtained. In fact, TMS indexes, especially when registering from speech related muscles, may be influenced by peripheral activity and muscular activation. Moreover, the here reported qualitative differences in correlations between groups (for example in pre-TMS EMG; see S1 File), sometimes have to be cautiously interpreted, considering the different sample sizes. Indeed, difficulties in recruiting a homogenous sample of DS participants should always be considered [120]. When considering DS, especially in adulthood, it is always difficult to disentangle between causal neural mechanisms (perhaps related to factors that predispose to the development of the disturbance), long-life stuttering (and its neural consequences), and compensatory neural patterns, both at cortical and spinal levels. This is quite common in movement disorders, such as Parkinson’s Disease or dystonia. In fact, neural activations may be related to maladaptive plasticity as a consequence of a life of stuttering. This might be especially true when considering that abnormal/defective sensory inputs (as for example those related to stuttering episodes [121]) may be related to deviant neural motor activations [122], acting also on neural plasticity. This may finally result in a cascade of neural changes that help to maintain the disorder. For these reasons, the present findings cannot automatically be generalized to DS in childhood.

## Conclusions and Future Perspectives

The present work contributes to defining the aberrant balance between motor excitatory and inhibitory mechanisms in tongue representation of the primary motor cortex of DS adults, even when no overt dysfluency is occurring (i.e. in a stuttering “*state*” condition) [5,13]. It generally confirms that motor (excitatory/inhibitory) balance is a fundamental issue when considering neural functioning in motor disorders [123]. It has been conducted during no concurrent speech tasks, to better investigate some previously undefined indexes of intracortical motor excitability in DS (tongue silent period). The use of a “*basic*” functional modality opens the way toward more complex TMS studies aimed at investigating modulations of aberrant indexes during complex speech and motor tasks. The present findings confirm that DS is a motor disturbance characterized by an abnormal ratio between excitatory and inhibitory neural circuits, especially in the speech motor networks of the left hemisphere, but suggest tongue silent period as a novel neural marker for persistent DS, also in relation to here reported relations with stuttering severity. In fact, silent period may reflect neural oscillations in cortical (motor) systems, reflecting excitatory/inhibitory ratios [124]. Dysfluency could be only the more evident symptom of a subtle motor syndrome, characterized by the constant presence of an aberrant excitability of motor system and an aberrant interplay between its components. The present data will be useful to help define more focused neural targets for disentangling new treatment options, such as non-invasive neuro-modulation or more focused pharmacological interventions, targeting and modulating here reported indexes of abnormal functioning in persistent DS.

## Supporting Information

S1 File (PDF)

## References

[1] Mulligan HF, Anderson TJ, Jones RD, Williams MJ, Donaldson IM. Tics and developmental stuttering. Park Relat Disord. 2003;9: 281–289. 10.1016/S1353-8020(03)00002-6 12781595

[2] Riva-Posse P, Busto-Marolt L, Schteinschnaider A, Martinez-Echenique L, Cammarota A, Merello M. Phenomenology of abnormal movements in stuttering. Park Relat Disord. 2008;14: 415–419. 10.1016/j.parkreldis.2007.11.006 18316236

[3] Alm PA. Stuttering and the basal ganglia circuits: a critical review of possible relations. J Commun Disord. 2004;37: 325–369. 10.1016/j.jcomdis.2004.03.001 15159193

[4] Craig-McQuaide A, Akram H, Zrinzo L, Tripoliti E. A review of brain circuitries involved in stuttering. Front Hum Neurosci. 2014;8: 884 10.3389/fnhum.2014.00884 25452719 PMC4233907

[5] Neef NE, Anwander A, Friederici AD. The neurobiological grounding of persistent stuttering: from structure to function. Curr Neurol Neurosci Rep. 2015;15: 63 10.1007/s11910-015-0579-4 26228377

[6] Fisher SE. Genetic susceptibility to stuttering. New Eng J Med. 2010;362: 750–752. 10.1056/NEJMe0912594 20147708

[7] Bloodstein O, Ratner NB. A handbook on stuttering. 6th ed Clifton Park (NY): Thomson Delmar; 2008.

[8] Cykowski MD, Fox PT, Ingham RJ, Ingham JC, Robin DA. A study of the reproducibility and etiology of diffusion anisotropy differences in developmental stuttering: a potential role for impaired myelination. NeuroImage. 2010;52: 1495–1504. 10.1016/j.neuroimage.2010.05.011 20471482 PMC4135434

[9] Kang C, Riazuddin S, Mundorff J, Krasnewich D, Friedman P, Mullikin JC, et al Mutation in the lysosomal enzyme-targeting pathway and persistent stuttering. New Eng J Med. 2010;362: 677–685. 10.1056/NEJMoa0902630 20147709 PMC2936507

[10] Sommer M, Koch MA, Paulus W, Weiller C, Büchel C. Disconnection of speech-relevant brain areas in persistent developmental stuttering. Lancet. 2002;360: 380–383. 10.1016/S0140-6736(02)09610-1 12241779

[11] Watkins KE, Smith SM, Davis S, Howell P. Structural and functional abnormalities of the motor system in developmental stuttering. Brain. 2008;131: 50–59. 10.1093/brain/awm241 17928317 PMC2492392

[12] Brown S, Ingham RJ, Ingham JC, Laird AR, Fox PT. Stuttered and fluent speech production: an ALE meta-analysis of functional neuroimaging studies. Hum Brain Mapp. 2005;25: 105–117. 10.1002/hbm.20140 15846815 PMC6871755

[13] Budde KS, Barron DS, Fox PT. Stuttering, induced fluency, and natural fluency: a hierarchical series of activation likelihood estimation meta-analyses. Brain Lang. 2014;139: 99–107. 10.1016/j.bandl.2014.10.002 25463820 PMC4405378

[14] Ingham RJ, Grafton ST, Bothe AK, Ingham JC. Brain activity in adults who stutter: similarities across speaking tasks and correlations with stuttering frequency and speaking rate. Brain Lang. 2012;122: 11–24. 10.1016/j.bandl.2012.04.002 22564749 PMC3372660

[15] Wymbs NF, Ingham RJ, Ingham JC, Paolini KE, Grafton ST. Individual differences in neural regions functionally related to real and imagined stuttering. Brain Lang. 2013;124: 153–164. 10.1016/j.bandl.2012.11.013 23333668 PMC3625940

[16] Bosshardt HG. Effects of concurrent cognitive processing on the fluency of word repetition: comparison between persons who do and do not stutter. J Fluency Disord. 2002;27: 93–113. 10.1016/S0094-730X(02)00113-4 12145987

[17] Daliri A, Prokopenko RA, Flanagan JR, Max L. Control and prediction components of movement planning in stuttering versus nonstuttering adults. J Speech Lang Hear Research. 2014;57: 2131–2141. 10.1044/2014_JSLHR-S-13-0333 25203459 PMC4270877

[18] Forster DC, Webster WG. Speech-motor control and interhemispheric relations in recovered and persistent stuttering. Dev Neuropsychol. 2001;19: 125–145. 10.1207/S15326942DN1902_1 11530972

[19] Smits-Bandstra S, De Nil LF. Sequence skill learning in persons who stutter: implications for cortico-striato-thalamo-cortical dysfunction. J Fluency Disord. 2007;32: 251–278. 10.1016/j.jfludis.2007.06.001 17963936

[20] Webster WG. Motor performance of stutterers: a search for mechanisms. J Mot Behav. 1990;22: 553–571. 15117662 10.1080/00222895.1990.10735528

[21] Wu J. C., Maguire G., Riley G., Fallon J., La Casse L., Chin S., et al A positron emission tomography [18F]deoxyglucose study of developmental stuttering. Neuroreport. 1995;6: 501–555. 10.1097/00001756-199502000-00024 7766852

[22] Wu JC, Maguire G, Riley G, Lee A, Keator D, Tang C, et al Increased dopamine activity associated with stuttering. Neuroreport. 1997;8: 767–770. 10.1097/00001756-199702100-00037 9106763

[23] Etchell AC, Johnson BW, Sowman PF. Behavioral and multimodal neuroimaging evidence for a deficit in brain timing networks in stuttering: a hypothesis and theory. Front Hum Neurosci. 2014;8: 467 10.3389/fnhum.2014.00467 25009487 PMC4070061

[24] Boldrini M, Rossi M, Placidi GF. Paroxetine efficacy in stuttering treatment. Int J Neuropsychopharmacol. 2003;6: 311–312. 10.1017/S1461145703003584 12975000

[25] Busan P, Battaglini PP, Borelli M, Evaristo P, Monti F, Pelamatti G. Investigating the efficacy of paroxetine in developmental stuttering. Clin Neuropharmacol. 2009;32: 183–188. 10.1097/WNF.0b013e31819817eb 19620850

[26] Kumar A, Balan S. Fluoxetine for persistent developmental stuttering. Clin Neuropharmacol. 2007;30: 58–59. 10.1097/01.wnf.0000240950.18821.19 17272973

[27] Maguire GA, Riley GD, Franklin DL, Gottschalk LA. Risperidone for the treatment of stuttering. J Clin Psychopharmacol. 2000;20: 479–482. 10.1097/00004714-200008000-00013 10917410

[28] Maguire GA, Riley GD, Franklin DL, Maguire ME, Nguyen CT, Brojeni PH. Olanzapine in the treatment of developmental stuttering: a double-blind, placebo-controlled trial. Ann Clin Psychiatry. 2004;16: 63–67. 10.1080/10401230490452834 15328899

[29] Maguire G, Franklin D, Vatakis NG, Morgenshtern E, Denko T, Yaruss JS, et al Exploratory randomized clinical study of pagoclone in persistent developmental stuttering: the examining pagoclone for persistent developmental stuttering study. J Clin Psychopharmacol. 2010;30: 48–56. 20075648 10.1097/JCP.0b013e3181caebbe

[30] Tavano A, Busan P, Borelli M, Pelamatti G. Risperidone reduces tic-like motor behaviors and linguistic dysfluencies in severe persistent developmental stuttering. J Clin Psychopharmacol. 2011;31: 131–133. 10.1097/JCP.0b013e318205694f 21192161

[31] Busan P, D’Ausilio A, Borelli M, Monti F, Pelamatti G, Pizzolato G, et al Motor excitability evaluation in developmental stuttering: a transcranial magnetic stimulation study. Cortex. 2013;49: 781–792. 10.1016/j.cortex.2011.12.002 22225881

[32] Ingham RJ, Fox PT, Ingham JC, Xiong J, Zamarripa F, Hardies LJ, et al Brain correlates of stuttering and syllable production: gender comparison and replication. J Speech Lang Hear Res. 2004;47: 321–341. 10.1044/1092-4388(2004/026) 15157133

[33] Walsh B, Mettel KM, Smith A. Speech motor planning and execution deficits in early childhood stuttering. J Neurodev Disord. 2015;7: 27 10.1186/s11689-015-9123-8 26300988 PMC4545974

[34] Inghilleri M, Conte A, Currà A, Frasca V, Lorenzano C, Berardelli A. Ovarian hormones and cortical excitability. An rTMS study in humans. Clin Neurophysiol. 2004;115: 1063–1068. 10.1016/j.clinph.2003.12.003 15066531

[35] Alm PA, Karlsson R, Sundberg M, Axelson HW. Hemispheric lateralization of motor thresholds in relation to stuttering. PLoS One. 2013;8: e76824 10.1371/journal.pone.0076824 24146930 PMC3795648

[36] Barwood CHS, Murdoch BE, Goozee JV, Riek S. Investigating the neural basis of stuttering using transcranial magnetic stimulation: preliminary case discussions. Speech Lang Hear. 2013;16: 18–27. 10.1179/2050571X12Z.0000000001

[37] Neef NE, Hoang TN, Neef A, Paulus W, Sommer M. Speech dynamics are coded in the left motor cortex in fluent speakers but not in adults who stutter. Brain. 2015;138: 712–725. 10.1093/brain/awu390 25595146 PMC4408433

[38] Neef NE, Jung K, Rothkegel H, Pollok B, von Gudenberg AW, Paulus W, et al Right-shift for non-speech motor processing in adults who stutter. Cortex. 2011;47: 945–954. 10.1016/j.cortex.2010.06.007 20822768

[39] Neef NE, Paulus W, Neef A, von Gudenberg AW, Sommer M. Reduced intracortical inhibition and facilitation in the primary motor tongue representation of adults who stutter. Clin Neurophysiol. 2011;122: 1802–1811. 10.1016/j.clinph.2011.02.003 21377925

[40] Rogić Vidaković M, Schonwald Zmajević M, Jurić T, Erceg N, Bubić A, Vulević Z. Evaluation of corticobulbar and corticospinal excitability in developmental stuttering: the navigated transcranial magnetic stimulation study. Conference: Brain Stimulation, at 1st International Brain Stimulation Conference 2–4 March 2015. 2015;8: 317–318.

[41] Sommer M, Knappmeyer K, Hunter EJ, von Gudenberg AW, Neef N, Paulus W. Normal interhemispheric inhibition in persistent developmental stuttering. Mov Disord. 2009;24: 769–773. 10.1002/mds.22383 19224611

[42] Sommer M, Wischer S, Tergau F, Paulus W. Normal intracortical excitability in developmental stuttering. Mov Disord. 2003;18: 826–830. 10.1002/mds.10443 12815664

[43] Rogić Vidaković M, Jerković A, Jurić T, Vujović I, Šoda J, Erceg N, et al Neurophysiologic markers of primary motor cortex for laryngeal muscles and premotor cortex in caudal opercular part of inferior frontal gyrus investigated in motor speech disorder: a navigated transcranial magnetic stimulation (TMS) study. Cogn Process, *in press* 10.1007/s10339-016-0766-5 27130564

[44] D’Ausilio A, Jarmolowska J, Busan P, Bufalari I, Craighero L. Tongue corticospinal modulation during attended verbal stimuli: priming and co-articulation effects. Neuropsychologia. 2011;49: 3670–3676. 10.1016/j.neuropsychologia.2011.09.022 21958646

[45] Rossi S, Hallett M, Rossini PM, Pascual-Leone A, The Safety of TMS Consensus Group. Safety, ethical considerations, and application guidelines for the use of transcranial magnetic stimulation in clinical practice and research. Clin Neurophysiol. 2009;120: 2008–2039. 10.1016/j.clinph.2009.08.016 19833552 PMC3260536

[46] Rossi S, Hallett M, Rossini PM, Pascual-Leone A. Screening questionnaire before TMS: an update. Clin Neurophysiol. 2011;122: 1686 10.1016/j.clinph.2010.12.037 21227747

[47] Oldfield RC. The assessment and analysis of handedness: the Edinburgh Inventory. Neuropsychologia. 1971;9: 97–113. 10.1016/0028-3932(71)90067-4 5146491

[48] Lang N, Hasan A, Sueske E, Paulus W, Nitsche MA. Cortical hypoexcitability in chronic smokers? A transcranial magnetic stimulation study. Neuropsychopharmacology 2008;33: 2517–2523. 10.1038/sj.npp.1301645 18059439

[49] Khedr EM, Ahmed MA, Mohamed KA. Motor and visual cortical excitability in migraineurs patients with or without aura: transcranial magnetic stimulation. Neurophysiol Clin/Clin Neurophysiol. 2006;36: 13–18. 10.1016/j.neucli.2006.01.007 16530139

[50] Rosenkranz K, Williamon A, Rothwell JC. Motor cortical excitability and synaptic plasticity is enhanced in professional musicians. J Neurosci. 2007;27: 5200–5206. 10.1523/JNEUROSCI.0836-07.2007 17494706 PMC6672373

[51] Beck AT, Steer RA, Brown GK. Manual for the Beck Depression Inventory-II. San Antonio (TX): Psychological Corporation; 1996.

[52] McGregor KM, Zlatar Z, Kleim E, Sudhyadhom A, Bauer A, Phan S, et al Physical activity and neural correlates of aging: a combined TMS/fMRI study. Behav Brain Res. 2011;222: 158–168. 10.1016/j.bbr.2011.03.042 21440574 PMC3713467

[53] Zlatar ZZ, Towler S, McGregor KM, Dzierzewski JM, Bauer A, Phan S, et al Functional language networks in sedentary and physically active older adults. J Int Neuropsychol Soc. 2013;19: 625–634. 10.1017/S1355617713000246 23458438 PMC3691286

[54] Murdoch BE, Barwood CHS, Goozee JV, Riek S, Lloyd D. Determining the optimal current direction of transcranial magnetic stimulation to induce motor responses in the tongue: a preliminary study of neurologically healthy individuals. Speech Lang Hear. 2013;16: 56–67. 10.1179/2050571X13Z.0000000009

[55] Hallett M, Chokroverty S. Magnetic stimulation in clinical neurophysiology. Elsevier; 2005.

[56] Kallioniemi E, Säisänen L, Könönen M, Awiszus F, Julkunen P. On the estimation of silent period thresholds in transcranial magnetic stimulation. Clin Neurophysiol. 2014;125: 2247–2252. 10.1016/j.clinph.2014.03.012 24725846

[57] Lo YL, Fook-Chong S. The silent period threshold as a measure of corticospinal inhibition. J Clin Neurophysiol. 2005;22: 176–179. 15933489

[58] Säisänen L, Pirinen E, Teitti S, Könönen M, Julkunen P, Määttä S, et al Factors influencing cortical silent period: optimized stimulus location, intensity and muscle contraction. J Neurosci Methods. 2008;169: 231–238. 10.1016/j.jneumeth.2007.12.005 18243329

[59] Riley G. The stuttering severity instrument for adults and children (SSI-4). 4th ed Austin (TX): PRO-ED; 2009.

[60] Vanryckeghem M, Brutten G. A comparative investigation of the BigCAT and Erickson S-24 measures of speech-associated attitude. J Commun Disord. 2012;45: 340–347. 10.1016/j.jcomdis.2012.06.001 22763013

[61] Sanavio E, Bertolotti G, Michielin P, Vidotto G, Zotti AM. CBA-2.0: Cognitive Behavioural Assessment 2.0: scale primarie: manuale. Firenze (IT): O.S., Organizzazioni Speciali; 1997.

[62] Faraway JJ. Extending the linear model with R: generalized linear, mixed effects and nonparametric regression models. London (UK): Chapman & Hall/CRC; 2006.

[63] West BT, Welch KB, Galecki AT. Linear mixed models: a practical guide using statistical software. London (UK): Chapman & Hall/CRC; 2006 10.1201/b17198

[64] McDonald JH. Handbook of biological statistics. 3rd ed Baltimore (Maryland): Sparky House Publishing; 2014.

[65] Nakagawa S. A farewell to Bonferroni: the problems of low statistical power and publication bias. Behav Ecol. 2004;15: 1044–1045. 10.1093/beheco/arh107

[66] Perneger TV. What’s wrong with Bonferroni adjustment. Brit Med J. 1998;316: 1236–1238. 9553006 10.1136/bmj.316.7139.1236 PMC1112991

[67] Benjamini Y, Hochberg Y. Controlling the false discovery rate: a practical and powerful approach to multiple testing. J Royal Stat Soc. 1995;57: 289–300.

[68] Borenstein M. Effect sizes for continuous data In: Cooper H, Hedges LV, Valentine JC, editors. The handbook of research synthesis and meta-analysis. New York: Russell Sage Foundation; 2009 pp. 221–237.

[69] Borenstein M, Hedges LV, Higgins JPT, Rothstein HR. Introduction to meta-analysis. Chichester (UK): Wiley; 2009 10.1002/9780470743386

[70] Cohen J. Statistical power analysis for the behavioral sciences. 2nd ed Hillsdale: Lawrence Erlbaum; 1988.

[71] Dunlop WP, Cortina JM, Vaslow JB, Burke MJ. Meta-analysis of experiments with matched groups or repeated measures designs. Psychol Methods. 1996;1: 170–177. 10.1037/1082-989X.1.2.170

[72] Fritz CO, Richler JJ, Morris PE. Effect size estimates: current use, calculations, and interpretation. J Exp Psychol Gen. 2012;141: 2–18. 10.1037/a0024338 21823805

[73] Glass GV, Mc Gaw B, Smith ML. Meta-analysis in social research. Beverly Hills: Russell Sage Foundation; 1981.

[74] Hedges LV, Olkin I. Statistical methods for meta-analysis. London: Academic Press; 1985.

[75] Braun AR, Varga M, Stager S, Schulz G, Selbie S, Maisog JM, et al Altered patterns of cerebral activity during speech and language production in developmental stuttering. An H2(15)O positron emission tomography study. Brain. 1997;120: 761–784. 10.1093/brain/120.5.761 9183248

[76] Fox PT, Ingham RJ, Ingham JC, Zamarripa F, Xiong JH, Lancaster JL. Brain correlates of stuttering and syllable production. A PET performance-correlation analysis. Brain. 2000;123: 1985–2004. 10.1093/brain/123.10.1985 11004117

[77] Fox PT, Ingham RJ, Ingham JC, Hirsch TB, Downs JH, Martin C, et al A PET study of the neural systems of stuttering. Nature. 1996;382: 158–161. 10.1038/382158a0 8700204

[78] Neumann K, Euler HA, von Gudenberg AW, Giraud AL, Lanfermann H, Gall V, et al The nature and treatment of stuttering as revealed by fMRI. A within- and between group comparison. J Fluency Disord. 2003;28: 381–409. 14643071 10.1016/j.jfludis.2003.07.003

[79] Preibisch C, Neumann K, Raab P, Euler HA, von Gudenberg AW, Lanfermann H, et al Evidence for compensation for stuttering by the right frontal operculum. NeuroImage. 2003;20: 1356–1364. 10.1016/S1053-8119(03)00376-8 14568504

[80] Chang SE, Horwitz B, Ostuni J, Reynolds R, Ludlow CL. Evidence of left inferior frontal-premotor structural and functional connectivity deficits in adults who stutter. Cereb Cortex. 2011;21: 2507–2518. 10.1093/cercor/bhr028 21471556 PMC3183422

[81] Chang SE, Erickson KI, Ambrose NG, Hasegawa-Johnson MA, Ludlow CL. Brain anatomy differences in childhood stuttering. NeuroImage. 2008;39: 1333–1344. 10.1016/j.neuroimage.2007.09.067 18023366 PMC2731627

[82] Chang SE, Kenney MK, Loucks TMJ, Ludlow L. Brain activation abnormalities during speech and non-speech in stuttering speakers. NeuroImage. 2009;46: 201–212. 10.1016/j.neuroimage.2009.01.066 19401143 PMC2693291

[83] Salmelin R, Schnitzler A, Schmitz F, Freund HJ. Single word reading in developmental stutterers and fluent speakers. Brain. 2000;123: 1184–1202. 10.1093/brain/123.6.1184 10825357

[84] Kell CA, Neumann K, von Kriegstein K, Posenenske C, von Gudenberg AW, Euler H, et al How the brain repairs stuttering. Brain. 2009;132: 2747–2760. 10.1093/brain/awp185 19710179

[85] Lu C, Peng D, Chen C, Ning N, Ding G, Li K, et al Altered effective connectivity and anomalous anatomy in the basal ganglia-thalamocortical circuit of stuttering speakers. Cortex. 2010;46: 49–67. 10.1016/j.cortex.2009.02.017 19375076

[86] Cai S, Tourville JA, Beal DS, Perkell JS, Guenther FH, Ghosh SS. Diffusion imaging of cerebral white matter in persons who stutter: evidence for network-level anomalies. Front Hum Neurosci. 2014;8: 54 10.3389/fnhum.2014.00054 24611042 PMC3920071

[87] Chang S–E, Zhu DC, Choo AL, Angstadt M. White matter neuroanatomical differences in young children who stutter. Brain. 2015;138:694–711. 10.1093/brain/awu400 25619509 PMC4339778

[88] Civier O, Kronfeld-Duenias V, Amir O, Ezrati-Vinacour R, Ben-Shachar M. Reduced fractional anisotropy in the anterior corpus callosum is associated with reduced speech fluency in persistent developmental stuttering. Brain Lang. 2015;143: 20–31. 10.1016/j.bandl.2015.01.012 25728013

[89] Connally EL, Ward D, Howell P, Watkins KE. Disrupted white matter in language and motor tracts in developmental stuttering. Brain Lang. 2014;131: 25–35. 10.1016/j.bandl.2013.05.013 23819900

[90] Kronfeld-Duenias V, Amir O, Ezrati-Vinacour R, Civier O, Ben-Shachar M. The frontal aslant tract underlies speech fluency in persistent developmental stuttering. Brain Struct Funct. 2016;221: 365–381. 10.1007/s00429-014-0912-8 25344925

[91] Xuan Y, Meng C, Yang Y, Zhu C, Wang L, Yan Q, et al Resting-state brain activity in adult males who stutter. PLoS One. 2012;7: e30570 10.1371/journal.pone.0030570 22276215 PMC3262831

[92] Chang SE, Zhu DC. Neural network connectivity differences in children who stutter. Brain. 2013;136: 3709–3726. 10.1093/brain/awt275 24131593 PMC3859219

[93] Jäncke L, Hänggi J, Steinmetz H. Morphological brain differences between adult stutterers and non-stutterers. BMC Neurol. 2004;4: 23 10.1186/1471-2377-4-23 15588309 PMC539354

[94] Kemerdere R, de Champfleur NM, Deverdun J, Cochereau J, Moritz-Gasser S, Herbet G, et al Role of the left frontal aslant tract in stuttering: a brain stimulation and tractographic study. J Neurol. 2016;263: 157–167. 10.1007/s00415-015-7949-3 26559819

[95] Kinoshita M, de Champfleur NM, Deverdun J, Moritz-Gasser S, Herbet G, Duffau H. Role of fronto-striatal tract and frontal aslant tract in movement and speech: an axonal mapping study. Brain Struct Funct. 2015;220: 3399–3412. 10.1007/s00429-014-0863-0 25086832

[96] Berardelli A, Rona S, Inghilleri M, Manfredi M. Cortical inhibition in Parkinson's disease. A study with paired magnetic stimulation. Brain. 1996;119: 71–77. 8624695 10.1093/brain/119.1.71

[97] Cantello R, Tarletti R, Civardi C. Transcranial magnetic stimulation and Parkinson’s disease. Brain Res Rev. 2002;38: 309–327. 11890979 10.1016/s0165-0173(01)00158-8

[98] Ziemann U, Tergau F, Bruns D, Baudewig J, Paulus W. Changes in human motor cortex excitability induced by dopaminergic and antidopaminergic drugs. Electroenceph Clin Neurophysiol. 1997;105: 430–437. 9448644 10.1016/s0924-980x(97)00050-7

[99] Giraud AL, Neumann K, Bachoud-Levi AC, von Gudenberg AW, Euler HA, Lanfermann H, et al Severity of dysfluency correlates with basal ganglia activity in persistent developmental stuttering. Brain Lang. 2008;104: 190–199. 10.1016/j.bandl.2007.04.005 17531310

[100] Toyomura A, Fujii T, Kuriki S. Effect of an 8-week practice of externally triggered speech on basal ganglia activity of stuttering and fluent speakers. NeuroImage. 2015;109: 458–468. 10.1016/j.neuroimage.2015.01.024 25595501

[101] Toyomura A, Fujii T, Kuriki S. Effect of external auditory pacing on the neural activity of stuttering speakers. NeuroImage. 2011;57: 1507–1516. 10.1016/j.neuroimage.2011.05.039 21624474

[102] Narayana S, Laird AR, Tandon N, Franklin C, Lancaster JL, Fox PT. Electrophysiological and functional connectivity of the human supplementary motor area. NeuroImage. 2012;62: 250–265. 10.1016/j.neuroimage.2012.04.060 22569543 PMC3381058

[103] Butler JE, Petersen NC, Herbert RD, Gandevia SC, Taylor JL. Origin of the low-level EMG during the silent period following transcranial magnetic stimulation. ClinNeurophysiol. 2012;123: 1409–1414. 10.1016/j.clinph.2011.11.034 22209661

[104] Inghilleri M, Berardelli A, Cruccu G, Manfredi M. Silent period evoked by transcranial stimulation of the human cortex and cervicomedullary junction. J Physiol. 1993;466: 521–534. 8410704 PMC1175490

[105] Poston B, Kukke SN, Paine RW, Francis S, Hallett M. Cortical silent period duration and its implications for surround inhibition of a hand muscle. Eur J Neurosci. 2012;36: 2964–2971. 10.1111/j.1460-9568.2012.08212.x 22775302 PMC3463678

[106] Classen J, Schnitzler A, Binkofski F, Werhahn KJ, Kim YS, Kessler KR, et al The motor syndrome associated with exaggerated inhibition within the primary motor cortex of patients with hemiparetic stroke. Brain. 1997;120: 605–619. 9153123 10.1093/brain/120.4.605

[107] Orth M. Transcranial magnetic stimulation in Gilles de la Tourette syndrome. J Psychosom Res. 2009;67: 591–598. 10.1016/j.jpsychores.2009.07.014 19913663

[108] Currà A, Romaniello A, Berardelli A, Cruccu G, Manfredi M. Shortened cortical silent period in facial muscles of patients with cranial dystonia. Neurology. 2000;54: 130–135. 10636138 10.1212/wnl.54.1.130

[109] Daliri A, Prokopenko RA, Max L. Afferent and efferent aspects of mandibular sensorimotor control in adults who stutter. J Speech Lang Hear Res. 2013;56: 1774–1788. 10.1044/1092-4388(2013/12-0134) 23816664 PMC3795963

[110] Kelly EM, Smith A, Goffman L. Orofacial muscle activity of children who stutter: a preliminary study. J Speech Hear Res. 1995;38: 1025–1036. 10.1044/jshr.3805.1025 8558872

[111] Max L, Gracco VL. Coordination of oral and laryngeal movements in the perceptually fluent speech of adults who stutter. J Speech Lang Hear Res. 2005;48: 524–542. 10.1044/1092-4388(2005/036) 16197270

[112] Poulos MG, Webster WG. Family history as a basis for sub-grouping people who stutter. J Speech Hear Res. 1991;34: 5–10. 2008081 10.1044/jshr.3401.05

[113] Suresh R, Ambrose N, Roe C, Pluzhnikov A, Wittke-Thompson JK, Ng MC, et al New complexities in the genetics of stuttering: significant sex-specific linkage signals. Am J Hum Genet. 2006;78: 554–563. 10.1086/501370 16532387 PMC1424690

[114] Alm PA, Risberg J. Stuttering in adults: the acoustic startle response, temperamental traits, and biological factors. J Commun Disord. 2007;40: 1–41. 10.1016/j.jcomdis.2006.04.001 16814317

[115] Bleek B, Montag C, Faber J, Reuter M. The five factor model of personality applied to persons who stutter and a matched control group. J Commun Disord. 2011;44: 218–222. 21163491 10.1016/j.jcomdis.2010.11.001

[116] Iverach L, O’Brian S, Jones M, Block S, Lincoln M, Harrison E, et al The five factor model of personality applied to adults who stutter. J Commun Disord. 2010;43: 120–132. 10.1016/j.jcomdis.2009.12.001 20070974

[117] Alm PA. Stuttering in relation to anxiety, temperament, and personality: review and analysis with focus on causality. J Fluency Disord. 2014;40: 5–21. 10.1016/j.jfludis.2014.01.004 24929463

[118] Davis S, Shisca D, Howell P. Anxiety in speakers who persist and recover from stuttering. J Commun Disord. 2007;40: 398–417. 10.1016/j.jcomdis.2006.10.003 17157866

[119] Craig A, Blumgart E, Tran Y. The impact of stuttering on the quality of life in adults who stutter. J Fluency Disord. 2009;34: 61–71. 10.1016/j.jfludis.2009.05.002 19686883

[120] Jones M, Gebski V, Onslow M, Packman A. Statistical power in stuttering research: a tutorial. J Speech Lang Hear Res. 2002;45: 243–255. 10.1044/1092-4388(2002/019) 12003508

[121] Daliri A, Prokopenko RA, Max L. Afferent and efferent aspects of mandibular sensorimotor control in adults who stutter. J Speech Lang Hear Res 2013;56: 1774–88. 10.1044/1092-4388(2013/12-0134) 23816664 PMC3795963

[122] Espay AJ, Morgante F, Purzner J, Gunraj CA, Lang AE, Chen R. Cortical and spinal abnormalities in psychogenic dystonia. Ann Neurol. 2006;59: 825–834. 10.1002/ana.20837 16634038

[123] Ridding MC, Sheean G, Rothwell JC, Inzelberg R, Kujirai T. Changes in the balance between motor cortical excitation and inhibition in focal, task specific dystonia. J Neurol Neurosurg Psychiatry 1995;59: 493–498. 10.1136/jnnp.59.5.493 8530933 PMC1073711

[124] Farzan F, Barr MS, Hoppenbrouwers SS, Fitzgerald PB, Chen R, Pascual-Leone A, et al The EEG correlates of the TMS induced EMG silent period in humans. NeuroImage. 2013;83: 120–134. 10.1016/j.neuroimage.2013.06.059 23800790 PMC4211432

