# Supplementary material for: Altered Modulation of Silent Period in Tongue Motor Cortex of Persistent Developmental Stuttering in Relation to Stuttering Severity
Source: PLoS One. 2016 Oct 6;11(10):e0163959. doi: 10.1371/journal.pone.0163959 (PMC5053488; doi:10.1371/journal.pone.0163959)
Supplement: S1 File — (PDF) [file pone.0163959.s001.pdf]

Supporting Information

Altered modulation of intracortical networks in tongue motor cortex of persistent developmental stuttering in relation to stuttering severity

Pierpaolo Busan[1*], Giovanni Del Ben[2], Simona Bernardini[3], Giulia Natarelli[4], Marco Bencich[2], Fabrizio Monti[5], Paolo Manganotti[5], and Piero Paolo Battaglini[2]

[1]IRCCS Fondazione Ospedale San Camillo, Venice, Italy

[2]B.R.A.I.N.Center for Neuroscience, Department of Life Sciences, University of Trieste, Trieste, Italy

[3] ABC Balbuzie Turin, Italy

[4] Department of Developmental and Social Psychology, University of Padua, Padua, Italy

[5] Department of Medical, Surgical and Health Sciences, University of Trieste, Trieste, Italy

*Corresponding Author:

e-mail: pbusan@units.it (PB)

Recruitment curves (supplementary analyses)

When considering pre-TMS EMG activity, findings show a possible interaction between groups, stimulated hemispheres and intensity of stimulation (t_{19}=1.760, P=0.09), suggesting a difference between DS and fluent speakers in pre-TMS EMG when stimulation is delivered in the LH at 125% MT (higher EMG activity in fluent speakers: t_{24}=2.178, P=0.04(*); *Glass's delta*=0.610). In this case, a positive relation with physical activity is evident in all subjects (left hemisphere TMS/tongue right side EMG: Γ=0.59; left hemisphere TMS/tongue left side EMG: Γ=0.64). See Table A.

Table A. Supplementary data by TMS and EMG.

Neurophysiologic index/Exp. group	**Stuttering LH**	**Stuttering RH**	**Fluent speakers LH**	**Fluent speakers RH**
**EMG 110% MT (V/sec)**	216.3 (168.9)	173.5 (85.9)	352.5 (316.9)	304.7 (317.1)
	303.7 (322.3)	251.2 (140.4)	336.4 (269.2)	399.1 (751.8)
**EMG 125% MT (V/sec)**	*190.8 (114.9)*	211.4 (120.8)	*390.0 (348.1)*	355.1 (434.4)
	201.7 (121.8)	303.4 (311.3)	*434.3 (397.9)*	344.1 (552.7)
**EMG 140% MT (V/sec)**	265.9 (221.0)	188.1 (129.0)	381.1 (413.3)	323.3 (404.7)
	235.3 (152.2)	238.5 (215.4)	384.0 (354.0)	337.4 (496.1)
**EMG silent period (V/sec)**	*2497.3 (2380.8)*	*2810.9 (2998.8)*	2691.3 (2543.6)	2948.8 (2841.9)
	3373.9 (2018.7)	*4199.2 (3309.0)*	3004.7 (2275.6)	2798.2 (2243.9)
**AMT FDI (%)**	35.8 (8.8)	36.3 (8.8)	NA	NA
**SPT FDI (%)**	36 (8.6)	36.8 (8.3)	NA	NA
**Silent period duration FDI (ms)**	87.7 (35.7)	80.6 (22.6)	NA	NA
**Silent period latency FDI (ms)**	220 (1.7)	22.5 (1.2)	NA	NA

Data are reported for DS *vs.* fluent speakers. Mean values are accompanied by standard deviations in brackets. Data are reported for right/left side of the tongue when considering corresponding pre-TMS EMG activity, SPd and latencies obtained from FDI muscle. Marginally significant differences are reported in *italic*; LH=left hemisphere, RH=right hemisphere.

Supplementary correlation analysis

BigCATin DS correlated with tongue AMT (bilaterally) and with left hemisphere tongue SPT (left hemisphere AMT: Γ=0.47; right hemisphere AMT: Γ=0.53; SPT: Γ=0.49). Itresulted positively relatedalso with bilateral AMT and SPT of FDI muscles (left hemisphere AMT: Γ=0.55; right hemisphere AMT: Γ=0.86; left hemisphere SPT: Γ=0.55; right hemisphere SPT: Γ=0.85).A positive relation was evident also between phobic reactions (IP/PH, CBA 2.0) and left hemisphere tongue silent period durations (Γ=0.37). Physical activity, in all participants (DS and fluent speakers), negatively correlated with tongue silent period durations obtained when stimulating the right hemisphere (tongue right side: Γ=-0.57). BigCAT resulted, in DS, in a positive relation with BDI-II (Γ=0.80), and with CBA 2.0 indexes such as social phobia (IP/2; Γ=0.82). It resulted also in a negative relation with indexes of extroversion (EPQ/R-E, CBA 2.0; Γ=-0.75). BDI-II resulted, in DS, in a positive relation with anxiety state (STAI-X1, CBA 2.0; Γ=0.59), and social phobia (IP/2, CBA 2.0; r=0.83). Anxiety state and trait (STAI-X1 and STAI-X2, CBA 2.0) positively correlated with social phobia in DS (IP/2, CBA 2.0; STAI-X1: Γ=0.48; STAI-X2: r=0.76). A positive relation was also evident, in DS, when considering QD (depressive symptoms; CBA 2.0) and phobic reactions (IP/F, CBA 2.0; r=0.61). A similar positive relation was evident between physical concomitants of DS (SSI-4) and higher arousal (QPF/R, CBA 2.0; r=0.78), as well as between physical concomitants of DS (SSI-4) and indexes of phobia (IP/F; r=0.61). Finally, a positive correlation was evident between SSI-4 and age of DS participants (Γ=0.66). When considering correlations on neurophysiologic data from a more qualitative point of view, it is mainly evident that pre-TMS EMG data resulted, in fluent speakers, more inter-related with respect to similar DS data: in fact, about 70% of data, when considering parametric statistics (about 60% of data, when considering non-parametric statistics), resulted significantly related in fluent speakers. About only 20% of data, when considering parametric statistics (about 26% of data, when considering non-parametric statistics), showed similar significant outputs in DS. Tongue silent period data and

MEPs data resulted significantly correlated in about 20% of cases in fluent speakers, when considering parametric statistics, and in about 15% of data, when considering non-parametric ones. On the other hand, about 12% of data resulted correlated in DS when considering parametric relations, while about 9% of data, resulted related when considering non-parametric statistics. Finally, MEPs amplitudes and areas correlated with their correspondent pre-TMS EMG in about 25% of fluent speakers data (parametric statistics), and in about 17% of them (non-parametric statistics), while in DS they resulted related in about 5% (parametric statistics), and 13% (non-parametric statistics) of data. All findings generally refer to positive relations, which were more evident in the fluent speakers group.

Fig A. Motor evoked potentials obtained from recruitment curves.

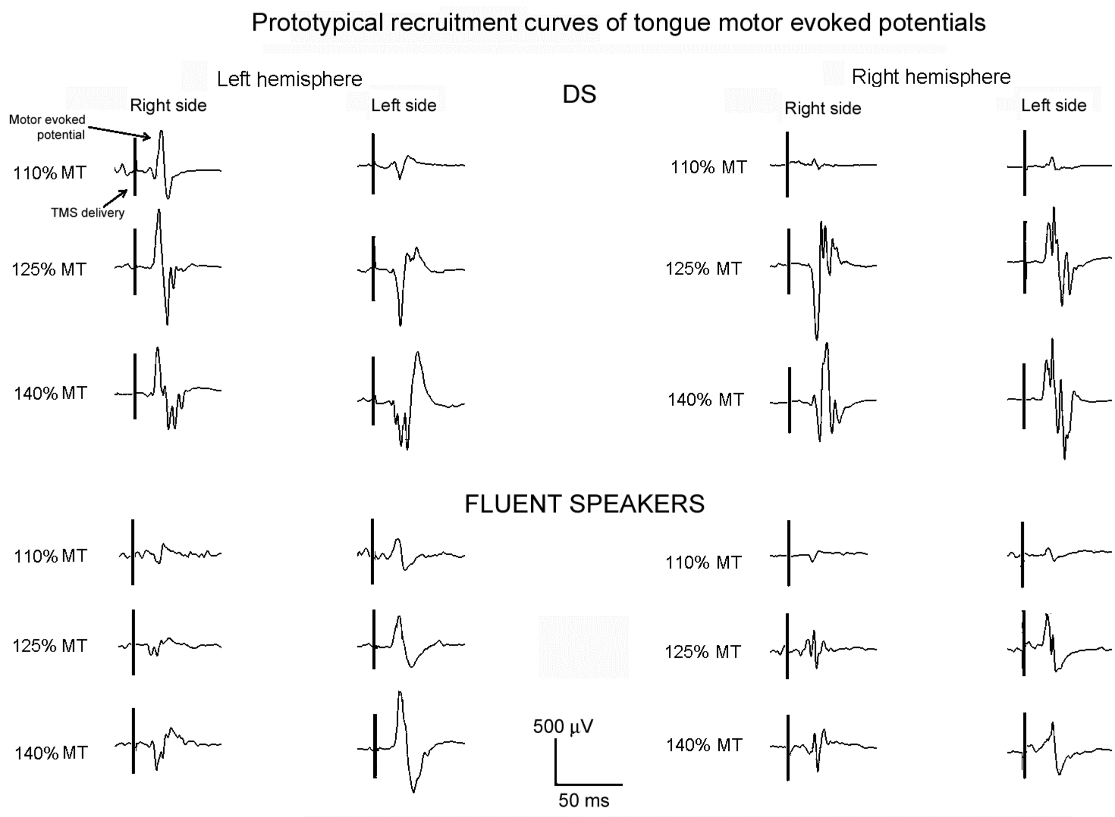

Recordings of raw, prototypical, single-trial motor evoked potentials, obtained from different subjects when delivering TMS and recording from tongue. Data are reported in DS and fluent speakers, for every condition. Data also illustrate inter- and intra-subject variability: increase of activation is not always linearly related to the increase of stimulation.

Fig B. Silent periods in stuttering and fluent speakers.

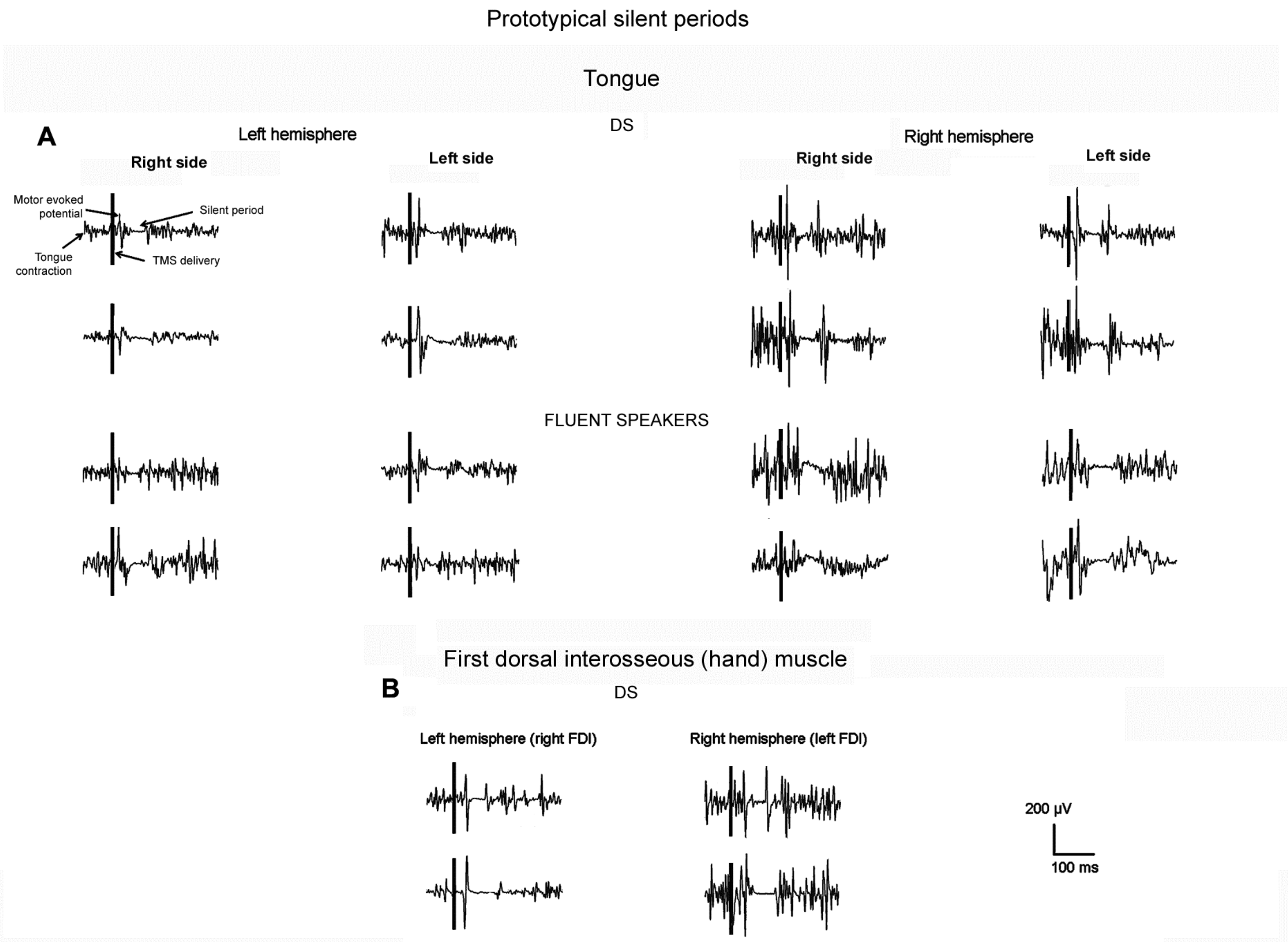

Tongue recordings of raw, prototypical, single-trial silent periods when delivering TMS. Data from different subjects are reported in DS and fluent speakers for every condition (A). Examples of silent periods obtained in DS from hand muscles are also reported (B). Data have been reported trying to consider also normal inter- and intra-subject variability.
